# Capsaicinoids supplementation decreases percent body fat and fat mass: adjustment using covariates in a post hoc analysis

**DOI:** 10.1186/s40608-018-0197-1

**Published:** 2018-08-13

**Authors:** James Rogers, Stacie L. Urbina, Lem W. Taylor, Colin D. Wilborn, Martin Purpura, Ralf Jäger, Vijaya Juturu

**Affiliations:** 1 Summit Analytical, LLC, 8354 Northfield Blvd., Building G, Suite 3700, Denver, CO 80238 USA; 2 Human Performance Laboratory, University of Mary Hardin-Baylor, Belton, TX 76513 USA; 3 Increnovo LLC, 2138 E Lafayette Pl, Milwaukee, WI 53202 USA; 4 OmniActive Health Technologies Inc., 67 East Park Place, Suite 500, Morristown, NJ 07950 USA

**Keywords:** Capsicum, Capsaicinoids, Statistical modelling, Body fat, Fat mass

## Abstract

**Background:**

Capsaicinoids (CAPs) found in chili peppers and pepper extracts, are responsible for enhanced metabolism. The objective of the study was to evaluate the effects of CAPs on body fat and fat mass while considering interactions with body habitus, diet and metabolic propensity.

**Methods:**

Seventy-five (*N* = 75) volunteer (male and female, age: 18 and 56 years) healthy subjects were recruited. This is a parallel group, randomized, double-blind, placebo controlled exploratory study. Subjects were randomly assigned to receive either placebo, 2 mg CAPs or 4 mg CAPs dosing for 12 weeks. After initial screening, subjects were evaluated with respect to fat mass and percent body fat at baseline and immediately following a 12-week treatment period. The current study evaluates two measures of fat loss while considering six baseline variables related to fat loss. Baseline measurements of importance in this paper are those used to evaluate body habitus, diet, and metabolic propensity. Lean mass and fat mass (body habitus); protein intake, fat intake and carbohydrate intake; and total serum cholesterol level (metabolic propensity) were assessed. Body fat and fat mass were respectively re-expressed as percent change in body fat and change in fat mass by application of formula outcome = (12-week value – baseline value) / baseline value) × 100. Thus, percent change in body fat and change in fat mass served as dependent variables in the evaluation of CAPs. Inferential statistical tests were derived from the model to compare low dose CAPs to placebo and high dose CAPs to placebo.

**Results:**

Percent change in body fat after 12 weeks of treatment was 5.91 percentage units lower in CAPs 4 mg subjects than placebo subjects after adjustment for covariates (*p* = 0.0402). Percent change in fat mass after 12 weeks of treatment was 6.68 percentage units lower in Caps 4 mg subjects than placebo subjects after adjustment for covariates (*p* = 0.0487).

**Conclusion:**

These results suggest potential benefits of Capsaicinoids (CAPs) on body fat and fat mass in post hoc analysis. Further studies are required to explore pharmacological, physiological, and metabolic benefits of both chronic and acute Capsaicinoids consumption.

**Trial registration:**

ISRCTN10458693 ‘retrospectively registered’.

## Background

Since approximately 7500 BC, chili peppers belonging to the species *Capsicum annuum* have been a part of the human diet in South, Middle, and North America. The plants were domesticated between 5200 and 3400 BC in U.S. and used as a food preserving substance in Mexico [1]. In U.S. consumption of all peppers has increased, rising from an average of 15.3 pounds per person in 2005 to 19.1 pounds per person in 2012 and consumption of bell peppers grew from 9.2 pounds to 11.7 pounds, while chili pepper consumption grew from 6.1 pounds to 7.4 pounds [2]. Red/Chili Peppers are widely cultivated in South America, Asia, Africa, and Mediterranean countries [3]. Pure Capsaicin measures 16,000,000 Scoville heat units (SHU). The spicy varieties of Capsicum are commonly called chili peppers, or simply “chilies”.

Capsicum (*Capsicum annuum* L. or *Capsicum frutescens* L.) and paprika (*Capsicum annuum* L.) are among the spices and other natural seasonings and flavorings that are generally recognized as safe (GRAS) for their intended use in food [4]. Capsicum and paprika are also listed among the essential oils, oleoresins (solvent-free), and natural extractives (including distillates) that are GRAS for their intended use in food [5, 6]. Capsaicinoids are mainly ingested as naturally occurring pungency-producing components of capsicum spices (chili, cayenne pepper, red pepper). The bell pepper is the only member of the *Capsicum* genus that does not produce capsaicin, a lipophilic chemical that can cause a strong burning sensation when it comes in contact with mucous membranes. The lack of capsaicin in bell peppers is due to a recessive form of a gene that eliminates capsaicin and, consequently, the “hot” taste usually associated with the rest of the *Capsicum* genus.

Parrish [7] reported that CAPs typically range from 0.10 mg/g in chili pepper to 2.50 mg/g in red pepper and 60 mg/g in red pepper oleoresin. In another study, Thomas et al. [8] reported that Capsicum varieties contain 0.22–20 mg total CAPs/g of dry weight. The amount of chili pepper used varies from country-to-country. For example, it was reported that the mean daily consumption of chili peppers in Mexico, Korea, Thailand, India and the United States are 15, 8, 5, 2.5, and 0.05–0.50 g/person/day, respectively [9]. Assuming that 1 g of chili contains 3 mg CAPs, the intake of CAPs in Mexico, Korea, Thailand, India and the United States will be approximately 45, 24, 15, 7.5, and 0.15–1.5 mg/person/day, respectively. The 2012 data from US indicate chili pepper consumption to be 9 g/person (CAPs consumption will be approximately 27.00 mg/person/day).

**Fig. 1:**
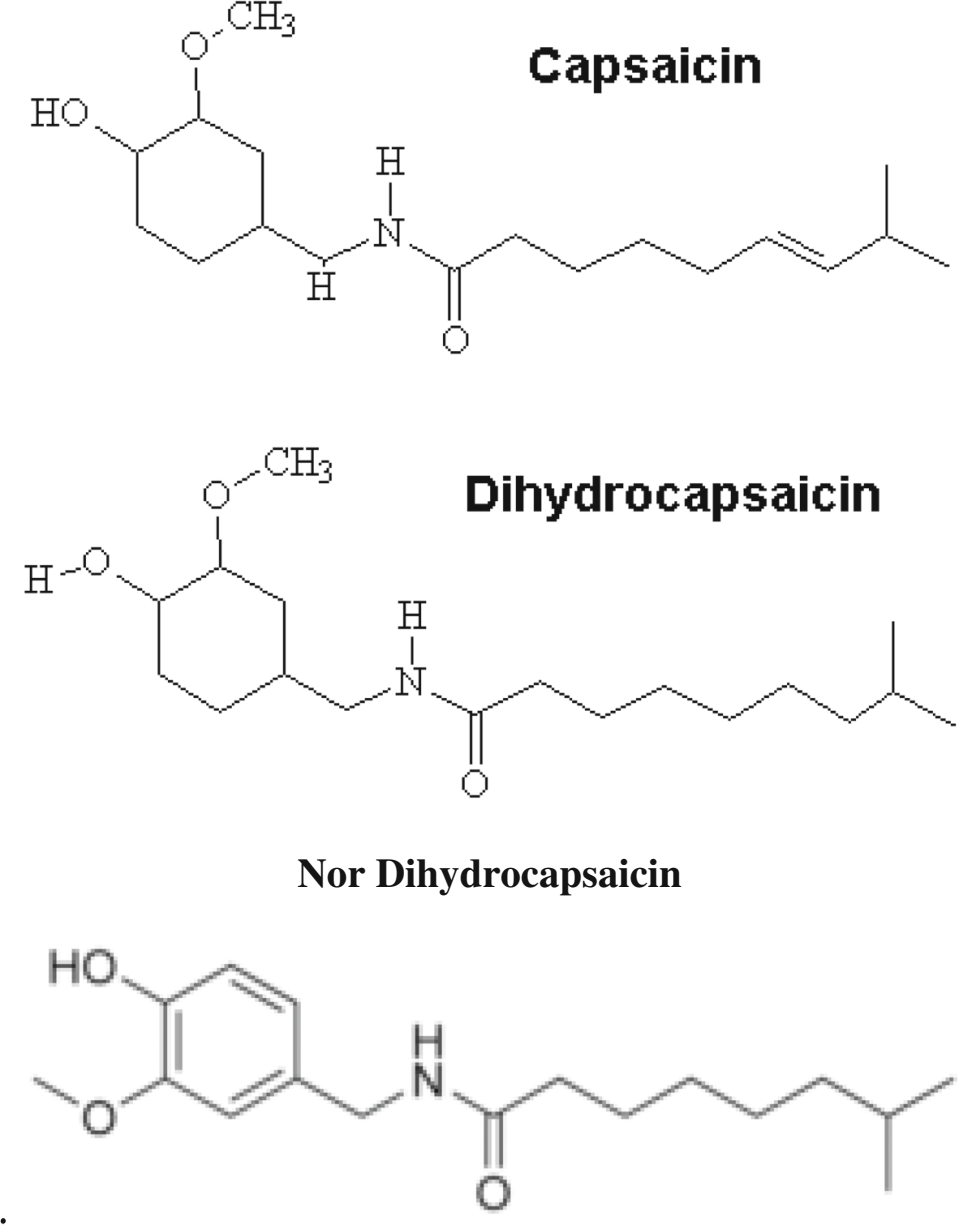
Components of Capsaicinoids from Capsicum extract

Capsaicinoids (CAPs, Fig. 1) are the major pungent, naturally occurring active compounds in chilli peppers [10, 11]. The available information indicates that CAPs possess a wide variety of biological and physiological activities, including neuropathic pain, inflammation, [12], reducing oxidative stress [13], antilithogenic effect, diabetic neuropathy, psoriasis, cardio protective, arthritis, and cancer [14]. Whiting et al. [15] indicated that CAPs play a beneficial role, as part of a weight management program. Capsaicinoids may have potential benefits on weight loss, lipolysis and stimulates thermogenesis and energy burning by activating receptors. These receptors include white and brown fat cells. The weight loss benefits of capsaicinoids are at the transient receptor potential cation channel subfamily V member 1, which is also known as vanilloid 1 or TRPV1. Whiting [15] met analysis showed that CAPs ingestion prior to a meal reduced ad libitum energy intake by 309.9 kJ (74.0 kcal) *p* < 0.001 during the meal. These results should be viewed as heterogeneity was high (I (2) =75.7%). Study findings suggest a minimum dose of 2 mg of CAPs may contribute to reductions in ad libitum energy intake. The molecular metabolic signaling mechanisms are by influencing metabolic rate, findings demonstrate CAPs appear to regulate hunger and satiety, blood metabolites, and catecholamine release [11, 15].

Weight management strategies consist of therapeutic lifestyle changes including increased physical activity and reduced caloric intake; however, some of these cases fail and/or significant weight loss is maintained only in the short term because of lack of compliance and potential weight recycling. Natural weight management ingredients aid weight reduction and compliance due to faster weight loss and increasing compliance. Weight management is known to be a complex function of genetic, metabolic, and behavioral components. It was theorized that in the absence of extremely large sample sizes, models taking these factors and the interactions between them into account would be needed to properly evaluate the impact of CAPs from Capsicum extract. Three background factors were thought to be of prima facie merit, namely, baseline body habitus, baseline diet and baseline metabolic propensity. The causes of the obesity epidemic are undoubtedly multifactorial [16, 17]. Recent research suggest body composition variables by covariate factors revealed 35, 28 and 21% of percentage of body fat (PBF), fat mass index (FMI), and fat free mass index (FFMI), respectively [18] and weight gain [19]. Baseline covariates include food intake, physical activity, alterations in sleep patterns, and the stresses impact the outcome in many clinical trials. Conducting exploratory analyses including such variables when large baseline imbalances are observed might be helpful to assess the robustness of the primary analysis.

This study investigates the impact on fat loss of CAPs, a nutraceutical specifically designed to facilitate the loss of fat. However, this is accomplished through the lens of a statistical model designed to control three prima facie background dimension of known relevance to fat loss. It was felt that this exploratory study, without facilitation using such a model, might from the beginning be doomed to failure due to lack of statistical power. By modeling the data, a type II error (i.e., the false denial of a supportive treatment outcome) is lessened.

## Methods

This is a parallel group, randomized, double-blind, placebo controlled study conducted at University of Mary Hardin-Baylor, TX, USA. Subjects included 54 Caucasians (34 female/20 male), 13 Hispanics (8 female/5 male), 8 African American (4 female/4 male) and 2 Asians (1 female/1 male). Subjects were evaluated with respect to percent fat mass and body fat at baseline and immediately following a 12-week treatment period. Subjects were randomly assigned to receive either placebo (Corn starch,), 2 mg CAPs dosing [100 mg Capsimax providing 2 mg capsaicinoids] or 4 mg CAPs dosing (100 mg × 2 Capsimax providing 4 mg capsaicinoids). Thus, this study employed a pretest – posttest design with three between subject conditions created by random assignment. Baseline measurements were prior to dosing and therefore qualify as covariates free of conflation with treatment. All experimental protocols were approved by the University of Mary Hardin-Baylor Institutional Review Board prior to initiation of research activities (ISRCTN registry #10458693).

### Subjects

The subjects were recruited based on paper advertisements, via flyers, telephone (verbal guide), email, social media, and internet targeting research participants to determine their eligibility and interest. Subjects were recruited who exhibited the following study inclusion characteristics: 1) male or female volunteers ranging between 18 and 56 years of age; 2) healthy; 3) no ergogenic supplement ingestion in the last 6 months; 4) able to comply with required study activities; 5) expressing agreement to avoid strenuous activity 24–48 h prior to study visits; 6) expressing agreement to avoid smoking, caffeine use and tobacco use for 12-h prior to study visits; 6) exhibiting a BMI between of 24.5–29.5 kg/m^2^; and 7) able to provide a written and dated informed consent for study participation.

Subjects were excluded from the study on the basis of the following characteristics: 1) consumption of ergogenic levels of nutritional supplements that may affect muscle mass or aerobic capacity (e.g., creatine, HMB, etc) or anabolic/catabolic hormones (e.g., androstenedione, DHEA, etc.) within 6 months of study start; 2) presence of any absolute or relative contraindication regarding exercise testing or study prescription as outlined by the ACSM; 3) reporting of any unusual adverse events associated with the study that in consultation with the supervising physician would results in recommended study removal; 4) presence of strong history of food or drug allergy of any kind; 5) ingestion of any dietary supplement (excluding multivitamins) within 1 month of study start; 6) existence of any chronic disease and or condition(s) that the principal investigator believes may jeopardize the study; or 7) existing pregnancy prior to or during the study.

There were 28 placebo subjects, 27 subjects in CAPs 2 mg (low dose) treatment and 22 subjects in CAPs 4 mg (high dose) treatment group completed the study. Seventy five subjects completed the treatments. Inclusion and exclusion criteria were used to screen patients for study entrance but were not used for treatment assignment which was random.

### Variables

Multiple study parameters were collected over various time points during the study. These variables included a diet log, laboratory values, cardio-metabolic parameters, body composition and anthropometric measurements, adverse events, and QoL (quality of life) indices.

In this study, dietary supplementation of Capsicum for 12 weeks has shown to promote appetite suppression, which translated to reduced self-reported caloric intake after 12 weeks of supplementation. While Capsicum administration resulted in improved body circumferences in a main effects analysis, it did not apparently affect DEXA fat mass or fat-free mass in a statistically significant way [20]. The current study evaluates two measures of fat loss while considering six baseline variables related to fat loss. Baseline measurements of importance in this paper are those used to evaluate body habitus, diet and metabolic propensity. The following parameters *at baseline* were respectively used for these variable types: lean mass and fat mass (body habitus); protein intake, fat intake and carbohydrate intake (diet); and total serum cholesterol level (metabolic propensity).

It was determined that in the statistical modeling for fat loss in this study, body habitus would be captured as baseline lean mass and baseline fat mass; diet would be captured in three variables, namely, baseline protein intake, fat intake, and carbohydrate intake assessed through food frequency questionnaires; and metabolic tendency would be capture as baseline total serum cholesterol level. The author reasoned that if fat loss could be modeled using these variables along with treatment assignment, the model predicted outcome of weight loss as a function of treatment, while controlling for these background factors, would afford an assessment of the impact of CAPs on fat loss. Without taking background factors into account, evaluation of CAPs would be carried out under suboptimal conditions relative to the available sample size. This paper has both an empirical and a methodologic intent. Studies of economically feasible sizes with the goal of screening a panel of outcome variables for treatment signals are important venues of discovery. These formative studies narrow future investigative windows and generate data based hypotheses. As such, they are an important contribution to the scientific literature. When such studies address complex outcome variables (which often are the variables of greatest interest) such as fat loss, too often they are analyzed with statistical models best suited for large summative trials (e.g., Phase III trials). This paper illustrates the utility of a model based approach to discovery in studies of a moderate size. The value of statistical modeling that accounts for important concomitant factors in fat loss is illustrated in this paper by evaluating fat loss using a simple analysis of variance model without covariates or interactions and a generalized linear model that includes both of these features.

### Investigational product

Capsicum extract is a Capsaicinoids enriched standardized product obtained from dried red fruits of *Capsicum annuum* L. The Capsicum extract is standardized into bead lets form (Capsimax) with food grade carbohydrates that is useful for food applications. Capsimax is a faint pinkish white colored free flowing uniform spheroidal bead lets with spicy odor, characteristics of dried ripe fruits of Capsicum. The product contains a minimum of 2% Capsaicinoids. The product is standardized to 2% Capsaicinoids, of which 1.2–1.35% is Capsaicin, 0.6–0.8% is dihydrocapsaicin, and 0.1–0.2% is nor-dihydrocapsaicin. The final product contains 15–25% extract from capsicum, 45–55% sucrose and 30–35% cellulose gum coatings.

### Body composition and blood chemistries

Participants received a whole-body dual x-ray absorptiometry (DEXA) scan for body composition assessment at baseline and 12 weeks (Hologic Wi; Hologic Inc., Bedford, MA). Prior to their study evaluations, subjects fasted overnight. Participants had a venous blood drawn from their arm via standard phlebotomy techniques at baseline visit and 12 weeks. A panel of blood health markers (lipid profile, metabolic health markers and complete blood counts) was assessed by sending samples to a commercial laboratory (Quest Diagnostics, Irving, TX).

A power analysis was done on 25 subjects and 21 subjects per group yield a power of 0.85 and 0.81 in terms of body composition changes.

### Statistical modeling

Prior to implementation of a statistical model to evaluate fat loss it was conceptualized that subject variation on three background dimension should be addressed during the evaluation process. These were baseline body habitus, baseline diet and baseline metabolic propensity. The objective was to parsimoniously capture these dimensions in as few variables as possible. Baseline lean mass and fat mass were used to capture body habitus; baseline carbohydrate intake, fat intake and protein intake were used to capture baseline diet; and baseline total cholesterol level was used to capture baseline metabolic propensity. Once selected, these baseline variables were used as independent variables in the statistical model used to evaluate fat loss. Percent body fat and fat mass were respectively re-expressed as percent change in body fat and change in fat mass by application of formula outcome = (12-week value – baseline value) / baseline value) × 100. Thus, percent change in body fat and fat mass served as dependent variables in the evaluation of CAPs.

After considering potential interactions among the baseline covariates and treatment, a comprehensive evaluation model was defined that expressed percent change in body fat (or fat mass) as a function of the baseline covariates noted previously, the treatment main effect and interactions between treatment and covariates. Interactions were a key component of the model as these were thought to capture the complex interplay between background factors and weight loss. We determined that the baseline variables and interactions could reasonably be expected to impact fat loss. Taking them into account during the evaluation of a nutraceutical designed to facilitate fat loss therefore seemed sensible.

In summary, two identical statistical models were used to respectively assess percent change in body fat and fat mass. Each model contained 1) the treatment effect (placebo, low dose and high dose); 2) six baseline covariates (carbohydrate intake, fat intake, protein intake, fat mass, lean mass and total cholesterol value); and 3) the interaction of each of the six covariates with treatment.

The above generalized linear model was used to estimate mean values and standard errors for percent change in body fat and fat mass. Inferential statistical tests were derived from the model to compare low dose CAPs to placebo and high dose CAPs to placebo. Covariate adjustment was Type III (each effect adjusted for all others); the model was obtained using Restricted Maximum Likelihood Estimation; and the model solution was accomplished using Newton-Raphson iterations [21–23]. Individual models were fit to percent change in percent body fat and to percent change in fat mass. Normal model convergence was observed. Missing values were accommodated in a manner that is typical for the generalized linear model. If a covariate value was missing at baseline, the subject was not evaluated in the model. No attempt was made to estimate the missing baseline value. As noted above, there were 77 total subjects in the study. Missing baseline values were concentrated in two subjects. Therefore, the net effect of missing baseline values was that 75 of the 77 total patients were available to each of the two models used to evaluate fat loss. Twenty-six rather than 27 patients were available from the Capsimax 2 mg treatment; all 22 patients were available from the Capsimax 4 mg treatment; and 27 of the 28 patients were available from the placebo group. Otherwise, as is an advantage of the generalized linear model, any missing values in the dependent variable were accommodated by the variance-covariance matrix to obtain model predicted means (i.e., Least Squares (LS) Means) and associated standard errors.

Tables present basic comparisons between treatment groups for the baseline variables serving as covariates. Tables also show the predicted means and standard errors as well as the significance levels for each treatment vs. control contrast obtained from the generalized linear model used to adjust for background factors. Finally, a one-way Analysis of Variance model was used to evaluate three treatments “without” covariate adjustment. These results were used to provide a base of comparison that modeled the data without accounting for the background factors of known importance to fat loss that were included as covariates in the generalized linear model described above.

This paper provides a comparison between the results obtained when important background factors are and are not included in the modeling of outcome data that concern complex physiological response variables such as fat loss. To do this, adjustments for multiplicity of comparisons must be avoided. Then, the unaltered *p*-values from the model with and the model without covariate adjustment will be available for “direct” comparison. If the more comprehensive model has increased statistical power, the contrast p-value for the comprehensive model should be lower than for the model that eliminates covariates and their interactions with treatment.

### Baseline variables

Age, height, weight, BMI, waist circumference, hip circumference and waist to hip ratio were compared to show that the treatment groups were similar with respect to variables of obvious relevance to fat loss; systolic blood pressure, diastolic blood pressure, and calorie intake were compared as these variables, too, are often associated with a history of resistance to fat loss. Descriptive statistics across the treatment groups are presented in Table 1. Additionally, analyses were conducted to compare the three treatment groups on all baseline variables simultaneously (i.e., a multivariate analysis was conducted) as well as individually (i.e., a univariate analysis of variance was conducted on each baseline variable).

**Table 1:** Descriptive statistics at baseline by treatment group

	Capsimax 2 mg		Capsimax 4 mg		Placebo	
Baseline variables						
	N	Mean ± SD	N	Mean ± SD	N	Mean ± SD
Age	27	31.07 ± 12.02	22	28.86 ± 11.58	28	28.71 ± 10.57
Height	27	170.13 ± 9.49	22	170.17 ± 8.58	28	173.05 ± 11.11
Weight	27	79.02 ± 20.12	22	80.16 ± 16.80	28	83.51 ± 19.67
SBP	27	115.04 ± 14.75	22	117.95 ± 10.28	28	118.89 ± 10.02
DBP	27	70.22 ± 10.18	22	73.18 ± 8.02	28	72.89 ± 9.64
BMI	27	27.02 ± 5.88	22	27.42 ± 4.15	28	27.80 ± 6.01
Waist	25	86.84 ± 17.38	22	89.90 ± 12.44	25	94.58 ± 17.87
Hip	25	102.44 ± 17.35	22	106.83 ± 10.27	25	107.36 ± 12.85
WHR	25	0.85 ± 0.06	22	0.84 ± 0.08	25	0.88 ± 0.09
Covariates						
Lean Mass	27	50,234.82 ± 14,130.64	22	52,153.33 ± 12,140.28	28	53,581.51 ± 14,316.47
Fat Mass	27	20,570.47 ± 11,206.61	22	19,903.67 ± 9337.78	28	20,960.33 ± 11,799.70
Protein Intake	26	82.52 ± 38.95	22	100.32 ± 68.52	27	88.66 ± 39.97
Carbohydrate Intake	26	239.61 ± 287.44	22	186.27 ± 70.64	27	188.05 ± 80.82
Fat Intake	26	66.67 ± 24.71	22	76.99 ± 34.58	27	77.75 ± 45.28
Serum Total-C	27	173.33 ± 27.97	22	168.36 ± 37.87	28	178.93 ± 42.75

*Total-C* Total cholesterol; *BMI* Body mass index; *SBP* Systolic blood pressure; *DBP* Diastolic Blood Pressure; *WHR* Waist Hip Ratio

1) Main Effect Multivariate p (Wilks’ Lambda) = 0.7073 for Baseline Variables. Univariate Main Effect *p*-values in all instances were *p* > 0.05 and ranged from *p* = 0.3047 to *p* = 0.8695. 2) Main Effect Multivariate p (Wilks’ Lambda) = 0.0.8562 for Baseline Covariates. Univariate Main Effect p-values in all instances were *p* > 0.05 and ranged from *p* = 0.3540 to *p* = 0.9438. 3) Baseline covariates explained extraneous variance and increased statistical power. Covariates did not dramatically change mean outcomes. There were no baseline differences. 3) *N* Number of subjects

## Results

Results for comparisons at baseline are first presented. Thereafter results for percent change in body fat and fat mass are presented.

### Baseline characteristics and baseline covariates

Table 1 presents descriptive statistics across the three treatment groups. The three groups are quite similar on baseline variable values as well as baseline covariate values. Both multivariate and univariate analyses carried out on baseline variables and baseline covariates resulted in unremarkable statistical significance levels (*p* > 0.05 in all instances).

### Body fat anf fat mass

#### Percent change in percent body fat

To provide a baseline against which the modeled data analysis can be compared, Table 2 presents the findings of a both a one-way ANOVA without control for background variables and the full generalized linear model that accommodates covariates and treatment by covariate interactions. Without consideration of baseline characteristics there is little difference between low dose CAPs 2 mg and placebo (difference = 0.20, *p* = 0.99) and no significant difference was observed. On the other hand, high dose CAPs 4 mg subjects evidence an improvement in percent change in percent body fat (− 0.70) while the placebo group exhibited a deterioration in percent change in percent body fat (2.70). However, the difference between these (− 3.39) was not statistically significant. *P*-values respectively for the CAPs 2 mg vs. placebo contrast and the CAPs 4 mg vs. placebo contrast are p = 0.99 (extreme non- significance) and *p* = 0.20 (not significant).

**Table 2:** Percent change in percent body fat compared between active and placebo groups using model generated means (LS Means)

Contrast	Treatment	N	LS Mean (SE)	LS Mean Difference (SE)	*P* Value
Without Baseline Covariates					
Capsimax 2 mg vs Placebo	Capsimax 2 mg	27	2.68 (1.78)	0.20	0.9939
	Placebo	28	2.70 (1.75)		
Capsimax 4 mg vs Placebo	Capsimax 4 mg	22	− 0.70 (1.97)	3.39	0.2014
	Placebo	28	2.70 (1.75)		
With Baseline Covariates					
Capsimax 2 mg vs Placebo	Capsimax 2 mg	26	3.51 (2.09)	−1.47	0.6030
	Placebo	27	4.99 (2.28)		
Capsimax 4 mg vs Placebo	Capsimax 4 mg	22	− 0.92 (2.06)	− 5.91	0.0402
	Placebo	27	4.99 (2.28)		

Analysis “Without Baseline Covariates” was a One-way ANOVA model. Analysis “With Baseline Covariates” was a generalized linear model containing treatment, variates and treatment x covariate interactions. Two subjects were lost to the “With Baseline Covariates” analysis due to missing values at baseline

The basic directional findings are very similar in the modeled data found in the Table 2 “With Baseline Covariates” analysis but the guiding *p*-values from the model suggest that the intuitive interpretation given above for the analysis lacking control for baseline factors meets the traditional 0.05 significance level for the high dose CAPS 4 mg vs. placebo contrast. Table 2 presents the model predicted means and treatment vs. placebo contrasts. Due to the considerable reduction in the model generated standard errors for the means (i.e., due to attribution of error variance to the covariates used as control variables) and the modeled equalization of covariates at baseline, the significance levels are greatly improved (i.e., statistical power is improved). For example, the model adjusted difference between CAPs 4 mg and placebo is − 5.91 percentage units (*p* = 0.0402). If the two analyses in Table 2 exhibited markedly different pattern of mean differences, one might doubt the validity of the model. In this case, the intuitive approach using basic unadjusted ANOVA and the generalized linear model approach that captures baseline factors of known a priori importance to fat loss, provide the same substantive message, albeit the latter with markedly improved statistical power and statistical significance for the high dose vs. placebo contrast.

#### Percent change in fat mass

As would be expected, the results for percent change in fat mass parallel those for percent change in percent body fat. Table 3 shows the presence of a favorable difference between high dose 4 mg CAPs and placebo of about 4 percentage points but this difference fails to obtain statistical significance (difference = − 4.07, *p* = 0.20). A small directional difference exists between low dose CAPs and placebo but this difference is distantly non-significant (− 0.82, *p* = 0.79). When adjustment for covariates is carried out through the model, Table 3 reveals the same general pattern of outcome (little difference between low dose CAPs 2 mg and placebo but a substantial difference between CAPs 4 mg and placebo), but after adjustment for baseline factors, the high dose CAPs 4 mg vs. placebo contrasts is statistically significant (difference = − 6.68, *p* = 0.05).

**Table 3:** Percent change in fat mass compared between active and placebo groups using model generated means (LS Means)

Contrast	Treatment	N	LS Mean (SE)	LS Mean Difference (SE)	*P* Value
Without Baseline Covariates					
Capsimax 2 mg vs Placebo	Capsimax 2 mg	27	2.44 (2.14)	−0.82	0.7860
	Placebo	28	3.26 (2.10)		
Capsimax 4 mg vs Placebo	Capsimax 4 mg	22	−0.81 (2.37)	−4.07	0.2037
	Placebo	28	3.26 (2.10)		
With Baseline Covariates					
Capsimax 2 mg vs Placebo	Capsimax 2 mg	26	3.45 (2.79)	−2.43	0.5044
	Placebo	27	5.88 (2.76)		
Capsimax 4 mg vs Placebo	Capsimax 4 mg	22	−0.80 (2.37)	−6.68	0.0487
	Placebo	27	5.88 (2.76)		

Analysis “Without Baseline Covariates” was a One-way ANOVA model. Analysis “With Baseline Covariates” was a generalized linear model containing treatment, covariates and treatment x covariate interactions. Two subjects were lost to the “With Baseline Covariates” analysis due to missing values at baseline

## Discussion

The simpler analysis without adjustment for covariance points to the same substantive understanding of the impact of CAPs on fat loss as the results derived from the generalized linear model that adjusts for covariates. The difference is a substantial increase in statistical power using the model. If the results were distinctly different between the basic analysis and those found after complex adjustment, it would be necessary to carefully unravel the reasons for such a difference. A decision would need to be made concerning whether the model, in fact represented a plausible outcome or presented an artefactual finding. In this case, however, the same general finding is present by both methods of analysis, the difference being that adjustment for background factors has markedly increased statistical power. The reasonable conclusion is CAPs at the high dose reduces percent body fat and fat mass.

A second supportive feature of the analysis is the presence of a dose response. However, in both analyses, there is a small directional finding favoring CAPs 2 mg over placebo, but the difference fails to reach statistical significance, markedly so in the basic analysis with an improved level of significance in the adjusted analysis that nevertheless is still clearly greater than the 0.05 benchmark. Overall, however, the results are consistent and plausible. The worst outcome was evidenced by placebo.

Capsicum has been shown to help improve metabolism and hormone function [24], diabetes [25], and reduce insulin and leptin resistance [26]. Capsicum and CAPs have also been linked to cardiovascular health, endothelial function [27], LDL-cholesterol oxidation [28], stimulate energy expenditure [11, 29–31]. This thermogenic effect has been exploited for purposes of weight management. Capsaicinoids have been reported to reduce appetite [32–34], increase thermogenesis [25, 35–38], and increase lipolysis [25, 35, 36, 39], or changes in serum glycerol and free fatty acids [10, 11]. The thermogenic effect of Capsaicinoids is mediated, at least in part, by a Capsaicinoid ­ sensitive structure located in the rostral ventrolateral medulla [40]. Capsaicinoids treatment may also stimulate vasodilation [27], which may indirectly impact thermogenesis, as any resultant loss of heat may necessitate an increase in metabolism.

Ludy et al. [41] reported effects of red pepper on energy balance from a combination of metabolic and sensory inputs. It was also suggested that individuals may become desensitized to red pepper. Capsaicin’s effect on appetite suppression, analgesia and lipolysis are mediated in part by expression of multiple genes involved in the lipid catabolic pathway, including those involved in thermogenesis [i.e., UCP2] and may be due to vanilloid receptor subtype 1 (VR1) binding capsaicin [42–45].

The following section explores potential biological pathways for the study findings on fat loss and influence of diet and body habitus. The methodological lesson from the current study is that in complex processes involving behavior, social and physiological components, such as fat loss, it is very important to control for at least some of the important subject background factors that might contribute to the directionality of the outcome parameter. In this instance, it was reasoned prior to final analysis that diet, body habitus and metabolic tendency would be important considerations when assessing fat loss. The data base contained three variables directly related to diet (baseline protein, fat and carbohydrate intake), two factors directly related to baseline body habitus (lean mass and fat mass), and a single encompassing variable that at least in part should capture metabolic tendency, namely, total cholesterol. Upon controlling for these factors, the statistical power of the inferential testing procedure was greatly increased. It was shown that higher dose 4 mg CAPs positively influenced fat loss, and after adjustment for background factors of obvious importance to fat loss, a resulting favorable CAPs vs. placebo difference existed with a probability of a type I error (i.e., a false positive conclusion) of less than 0.05.

In our first analysis for the study of CAPs effect on body composition, repeated measures without baseline covariate adjustments did not provided statistical significance for body fat and fat mass [46]. In the current analysis, baseline covariate adjustment resulted in statistical significance for body fat and fat mass in 4 mg CAPs treatment. This outcome *fact* suggests that baseline covariate analysis should have been considered at the point of protocol development. However, that it was not in the current instance does not eliminate the importance of fitting an appropriate model after the fact, particularly in light of the *known* relationship of the covariates selected here to fat loss. Models leading to insight at any point are contributory. A case might be made that it should be common practice in studies involving complex outcomes with suspected interactions with predisposing factors, that methodologies that anticipate the need for modeling be described a priori. For example, it is possible to include meaningful covariates in the data capture process at the beginning of a study and to outline methods of model development that will use those covariates, even if the precise model ultimately used is not described in advance. The limitations of the study are the L-CAP and H-CAP groups were supplemented with 2 mg/d and 4 mg/d of capsaicinoids, and other studies have supplemented participants with much higher doses (i.e., 135 mg/d [6]). In addition, our participants were healthy and were mildly overweight but not obese. Thus, if capsaicinoid supplementation is indeed effective at improving body composition, then more double blind clinical studies need to be performed in participants with greater BMIs.

Whitting et al. [15] observed an increase in energy expenditure (50 kcal/day) with capsaicinoid consumption, and that this would produce clinically significant levels of weight loss in 1–2 years. It was also observed that regular consumption significantly reduced abdominal adipose tissue levels and reduced appetite and energy intake [15]. In a met analysis, it was observed that CAPs increased lipid oxidation (recorded by measuring respiratory gases) or a decrease in fat stores. Further clarification is needed in terms of the specific ‘doses’ needed to reduction in abdominal body fat, energy intake and lipolysis.

Evidence suggests that the worldwide obesity epidemic is likely to continue its rise and several factors influence weight and risk of obesity [47]. Non-modifiable risk factors such as life style changes, healthy eating patterns, reducing caloric intake, and physical activity help to achieve long term weight loss. In the U.S. more than 20.60% women and 9.70% men are using weight loss dietary supplement at some point in their life and spend about $2 billion a year on weight loss dietary supplements in pill form (tablets, capsules, and soft gels). The use of multivitamin multi minerals decreased, and trends in use of individual supplements varied and were heterogeneous by population subgroups [48–50]. CAPs have shown effects on appetite, WHR [45], energy expenditure [15] and lipolysis [10, 11]. Capsaicinoids ingestion prior to a meal reduced ad libitum energy intake by 309.9 kJ (74.0 kcal) *p* < 0.001 during a meal. In a recent meta- analysis, suggest that capsaicin or capsaicinoids or capsiate could be a new therapeutic approach in obesity promoting a negative energy balance and increased fat oxidation [51]. Capsaicin/Capsaicinoids induces apoptosis and inhibits adipogenesis in pre-adipocytes and adipocytes. Activation of the transient receptor potential vanilloid-1 channels may prevent adipogenesis and improves visceral fat remodeling through the up-regulation of connexin [46] (Cx43) and regulates fat metabolism [52–57].

## Conclusion

Overall, CAPs to be used as long-term, natural weight management aide. Further long-term placebo controlled randomized trials with high dose of CAPs are now needed to investigate these effects further.

## Abbreviations

CAPs: Capsaicinoids
Cx43: Connexin
DEXA: Dual x-ray absorptiometry
FFMI: Fat free mass index
FMI: Fat mass index
LDL: Low-Density Lipoprotein
PBF: Percentage of body fat
QoL: Quality of life
SHU: Scoville heat units
TRP: Transient receptor potential
UCP: Uncoupling protein
VR1: Vanilloid receptor subtype 1
